# UNet-like network fused swin transformer and CNN for semantic image synthesis

**DOI:** 10.1038/s41598-024-65585-1

**Published:** 2024-07-21

**Authors:** Aihua Ke, Jian Luo, Bo Cai

**Affiliations:** 1 School of Cyber Science and Engineering, Wuhan University, Wuhan, 430072 China; 2 Key Laboratory of Aerospace Information Security and Trusted Computing, Ministry of Education, Wuhan, 430072 China

**Keywords:** Mathematics and computing, Computer science

## Abstract

Semantic image synthesis approaches has been dominated by the modelling of Convolutional Neural Networks (CNN). Due to the limitations of local perception, their performance improvement seems to have plateaued in recent years. To tackle this issue, we propose the SC-UNet model, which is a UNet-like network fused Swin Transformer and CNN for semantic image synthesis. Photorealistic image synthesis conditional on the given semantic layout depends on the high-level semantics and the low-level positions. To improve the synthesis performance, we design a novel conditional residual fusion module for the model decoder to efficiently fuse the hierarchical feature maps extracted at different scales. Moreover, this module combines the opposition-based learning mechanism and the weight assignment mechanism for enhancing and attending the semantic information. Compared to pure CNN-based models, our SC-UNet combines the local and global perceptions to better extract high- and low-level features and better fuse multi-scale features. We have conducted an extensive amount of comparison experiments, both in quantitative and qualitative terms, to validate the effectiveness of our proposed SC-UNet model for semantic image synthesis. The outcomes illustrate that SC-UNet distinctively outperforms the state-of-the-art model on three benchmark datasets (Citysacpes, ADE20K, and COCO-Stuff) including numerous real-scene images.

## Introduction

Semantic layout is a label map obtained using segmentation techniques for the semantic understanding of real-scene images. Following the rapid development of semantic segmentation techniques on the field of computer vision, it has become easier to acquire semantic layout maps, which has led to higher research attention on semantic layout maps. The research tasks based on semantic layout mainly incorporate semantic image retrieval^1,2^, semantic image segmentation^3–5^, semantic image synthesis^6–8^, semantic image classification^9^ and semantic image annotation^10,11^. Semantic image synthesis is a special form of conditional image synthesis task, which aims to synthesize photo-realistic and well-aligned image conditioned on a given semantic layout. Semantic image synthesis has a wide range of practical applications, e.g., it allows ordinary users to control the scene image synthesis by simply modifying the semantic layout. In addition, it can be utilized as a data augmentation tool for deep model convergence by learning to synthesize fresh samples similar to the original image data.

**Figure 1:**
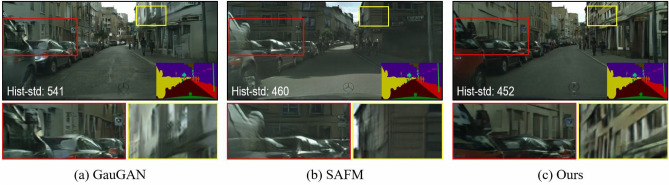
Visual comparison of the synthesized images produced by our method and other baseline approaches. Key differences are positioned with boxes on the synthesized image, and shown magnified below image. ‘Hist-std’ indicates the histogram’s standard deviation, where lower values indicate more balanced colors in the synthesized image.

Currently, pure CNN-based models have been the mainstream alternative for addressing the task of semantic image synthesis. CNN is proficient at exploiting the nature of local perception and weight sharing, which enables to extract local features of complex scene images at a low level and to reduce the training difficulty. Despite its many advantages, CNN is not fully exploited in semantic image synthesis. Since SwinT^12^ introduced the Swin Transformer to computer vision, it has achieved remarkable results in a wide range of downstream vision tasks, outperforming CNN in some cases. SwinT employs hierarchical vision transformers with shift windows to construct a backbone network, which can achieve a substantial improvement in synthesis performance on public datasets based on real scenes. zhang et al.^13^ extended the application of SwinT to image synthesis by proposing Styleswin. In order to actualise a broader receptive field, Styleswin utilizes the SwinT architecture to construct a hierarchical feature mapping relation. Despite the excellent success of Styleswin in modelling high-resolution image synthesis,it still suffers from some limitations in image synthesis tasks conditional on semantic layout: (1) The low-level features with the fine-grained information from the semantic layout are insufficiently utilized. (2) Overly weight-light decoder cannot efficiently fuse hierarchical semantic features at different scales.

To tackle the above limitations, this paper proposes the SC-UNet model, which is a UNet-like network fused Swin Transformer and CNN for semantic image synthesis. Our SC-UNet model adopts a UNet-like network structure composed of an encoder and a decoder, which can effectively extract and fuse the fine-grained features at the low level and the coarse-grained features at the high level, so as to achieve the higher synthesis performance. For the encoder part, we utilize a succession of hierarchical Swin Transformers with shift window as the basic unit of the network. The Swin Transformer-based encoder has the capability to efficiently extract low-level feature information at different scales from the input semantic layout map. To fully utilize the low-level features with fine-grained information, we introduce the skip connection before down-sampling with the patch merging layer to fuse the low-level positional information with the high-level semantic information. For the decoder part, the conventional linear projection or $1\times 1$ convolution can not sufficiently fuse the high- and low-level feature maps at different scales. Accordingly, we design a novel Conditional Residual Fusion (CRF) block, which as the classical structure of CNN can partially improve the synthesis performance. Moreover, the CRF block embeds an opposition-based learning mechanism and a weight assignment mechanism in the normalisation layer and the shortcut connection, respectively. Specifically, the opposition-based learning mechanism can efficiently augment semantic feature information through opposition-based learning, while the weight assignment mechanism can dynamically assign attentional weights over the channel and spatial dimensions.

Since our proposed SC-UNet method employs a supervised learning manner based on Generative Adversarial Networks (GAN), and utilizes the pre-trained Swin Transformer model to initialise the partial weights of the network. Therefore, the GAN-based SC-UNet approach reduces the occurrence probability of gradient disappearance or exploration owing to irrational initialisation. Figure 1 shows the visual comparison between the street scene images synthesised by our method and other baseline methods. From the figure, it can be clearly observed that our proposed SC-UNet method can synthesize more photo-realistic scene images by effectively mitigating common issues encountered in previous synthesis methods, such as local artifacts, color imbalance, and boundary ambiguities. Extensive experiment results, both qualitative and quantitative, conclude that the proposed SC-UNet method remarkably improves the performance of semantic image synthesis on three massive benchmark datasets, namely Cityscapes^14^, ADE20K^15^, and COCO-Stuff^16^. To enhance the robustness of our method, we sequentially resize the resolution of the three benchmark datasets to $256 \times 512$, $256 \times 256$ and $256 \times 256$.

The following are the contributions of our paper overall:

- We propose a UNet-like network model based on Swin Transformer and CNN for semantic image synthesis, which which overperforms the pure CNN-based model in effectively extracting high- and low-level features at different scales.
- We propose a decoder based on the Conditional Residual Fusion (CRF) block, which can produce more accurate feature representations through the hierarchical fusion of multi-scale features to improve the synthesis performance.
- We propose two novel mechanisms embedded in the CRF block, the opposition-based learning mechanism can effectively enhance the semantic feature information, while the weight assignment mechanism can dynamically assign attentional weights in channel and spatial dimensions.
- Extensive experiments are undertaken on three public datasets: Citysacpes, ADE20K and COCO-Stuff.The results prove the effectiveness of our semantic image synthesis method and the state of the art performance is achieved.

**Figure 2:**
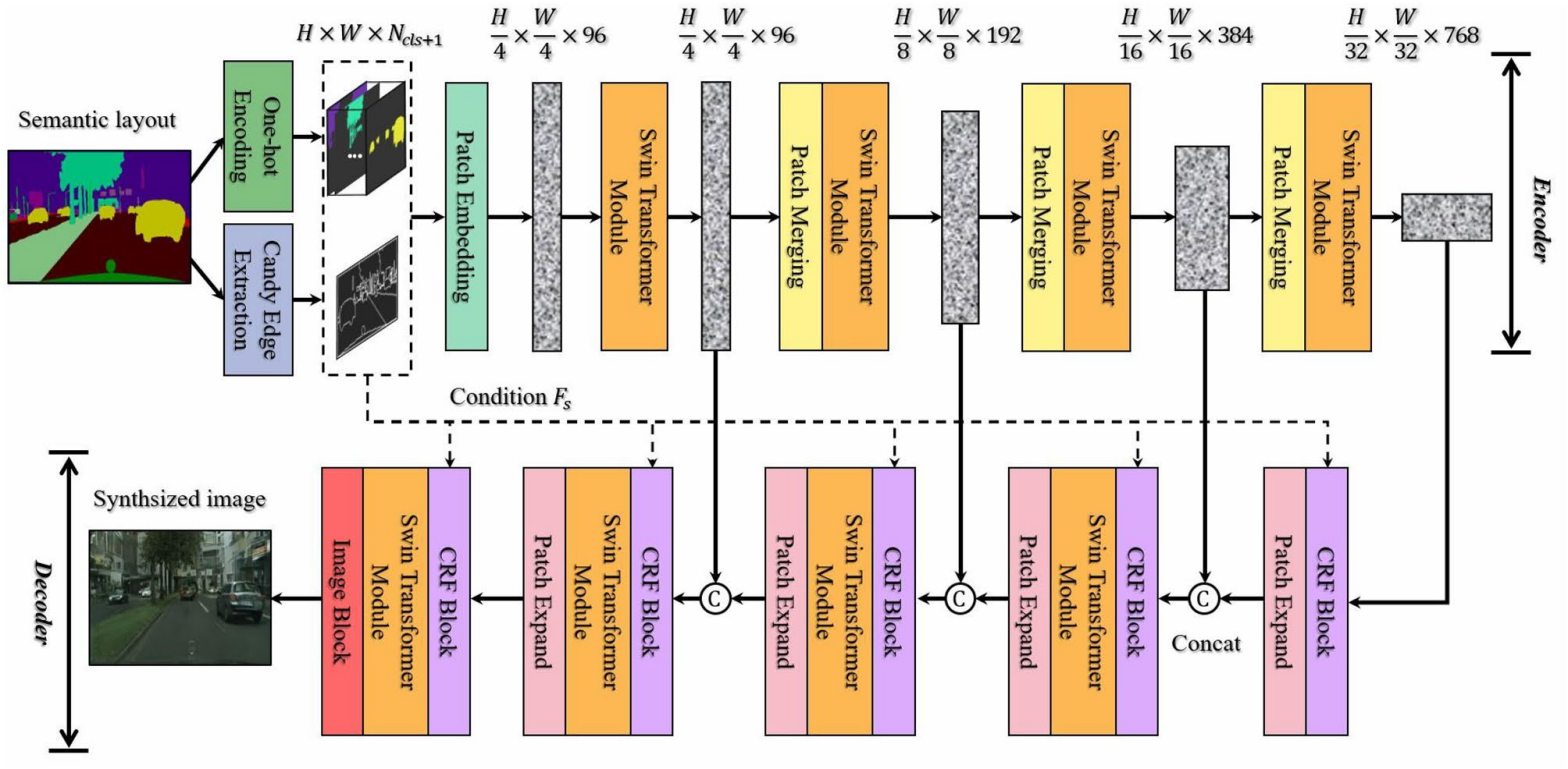
Overview of our approach SC-UNet.

## Related work

### Generative adversarial networks

Generative adversarial networks (GANs)^17,18^ have become the mainstream method for image synthesis tasks. GAN architecture is usually composed of two main networks, namely the generator and the discriminator. The generator is in charge of synthesizing the target images using the given input conditions. Nevertheless, the discriminator aims to distinguish between the synthetic image and the matched natural image. The input conditions used by GAN-based image synthesis methods are various, such as sparse sketches^19–21^, gaussian noise^22,23^, text descriptions^24–26^, natural images^27,28^, and semantic layout^29–32^. Considering the great success of GANs in image synthesis, we propose a novel GAN-based approach to tackle image synthesis conditioned only on semantic layout.

### Semantic image synthesis

Semantic image synthesis aims at generating high-fidelity image from the given semantic layout map. Such as, Pix2pix^33^ proposes a general-purpose solution for image-to-image translation problems by investigating conditional adversarial networks. Then, Pix2pixHD^30^ enhances it to achieve higher-resolution image synthesis from semantic label maps. Further, the normalization layer activations in GauGAN^22^ are modulated by affine transformations using the input semantic layout. Based on the improvement of this idea, CC-FPSE^34^ utilizes a semantic layout map as a condition to dynamically produce convolutional kernels. Similarly, both our approach and CC-FPSE are GAN-based semantic image synthesis models comprising generators and discriminators, utilizing features extracted from the semantic layout map to adaptively control the generation process. OASIS^32^ enhances the generator supervision by using feedback from spatial and semantic perceptual discriminators, thus eliminating the limitations of vgg-based perceptual loss on the above GAN model. Besides GAN models, CRN^29^ generates images with a photographic appearance that matches well with the input semantic layouts, and SIMS^35^ presents a semi-parametric approach to deal with the semantic image synthesis task by integrating the complementing benefits of parametric and non-parametric techniques. However, there is still opportunity for improvement in the quality of images generated using the previous state-of-the-art model. Therefore, we need to propose a novel method for more efficiently extracting the important information contained in a given semantic layout that may promote the development of semantic image synthesis.

### Conventional residual block

The conventional residual block, as a classical structure of convolutional neural network, has been extensively studied in prior research^36^. It typically consists of two convolutional layers and a shortcut connection, allowing for efficient transfer of input features to output features, thus facilitating cross-layer feature fusion. Additionally, the residual block helps mitigate the vanishing gradient problem and enhances network training capabilities. Kaiming et al.^36^ introduced a residual learning framework to train deeper networks effectively, paving the way for subsequent advancements. Ruofan et al.^37^ proposed a deep residual network for end-to-end projection learning, demonstrating its applicability in tasks involving Bayer images and high-resolution images. Despite the achievements of conventional residual blocks, they still exhibit limitations in image synthesis tasks conditioned on semantic layout maps. Recognizing this, we aim to augment traditional residual blocks by incorporating mechanisms inspired by Opposition-based Learning Mechanism (OLM) and Weight Assignment Mechanism (WAM). Where OLM is derived from the concept of opposition^38^, which aims to enhance learning by considering both positive and negative aspects of semantic features. This technique is employed to augment semantic information, thereby enhancing normalization performance. On the other hand, WAM can dynamically allocate attention weights on both channel and spatial dimensions. Despite previous research on similar mechanisms^39–41^ such as channel attention and spatial attention, WAM demonstrates uniqueness by integrating feature weighting, thereby showcasing innovation in image synthesis tasks. By integrating these components, we seek to enhance feature fusion and pay closer attention to semantic feature information, ultimately improving fusion performance in image synthesis tasks.

## Method

SC-UNet is a semantic image synthesis model based on a UNet-like network structure composed of an encoder and a decoder. The overall architecture of the SC-UNet model is shown in Fig. 2. In the encoding stage, the input semantic layout map is first semantically augmented by one-hot encoding operation and candy edge extraction operation, and then the augmented semantic features perform a patch embedding layer to obtain a sequence embedding for the input of the Swin Transformer module. Finally, the encoder based on Swin Transformer will extract the low-level features at different scales from the input sequence embedding. In the decoding stage, the decoder combined with the Conditional Residual Fusion (CRF) block and Swin Transformer module will hierarchically fuse the high-level semantic features with the low-level positional features. To recover the photo-realistic synthesized image with abundant details, our model finally employs a tanh activation function on the decoder’s output to maintain the pixel values within a specified range. Since our SC-UNet model utilizes a supervised training strategy based on Generative Adversarial Networks (GANs) and takes advantage of the pre-trained Swin Transformer module to initialize the partial weights of the network. Therefore, the GAN-based SC-UNet approach reduces the occurrence probability of exploding gradients owing to irrational initialisation. During the supervised training process, our model is optimised with the weighted summation of multiple loss functions, thus achieving better synthesis performance.

### Model encoder

The encoder of our proposed SC-UNet method aims to extract the low-level position features under different dimensions from the input semantic layout map. Let $M \in \mathbb {R}^{H \times W \times 1}$ be the input semantic layout map of the model, where *H* and *W* dimensions denote the height and width, respectively. To extract more accurate and comprehensive feature representations, the input semantic layout map first needs to be semantically augmented by simultaneously performing a one-hot encoding operation^15^ and a candy edge extraction operation. Among them, the one-hot encoding operation can map each object class in the semantic layout map with discrete nature into different channels, thus acquiring a more effective multi-channel feature representation. And the candy edge extraction operation can quickly and accurately extracts the positional information of the object edges from the input semantic layout map *M* by conducting several steps such as Gaussian blurring, gradient computation, non-maximum value suppression, double threshold detection and edge tracking, thus acquiring a feature representation for further processing by the encoder. Two feature representations resulted by augmenting the semantics are fused into a fresh feature representation $F_s \in \mathbb {R}^{H \times W \times N_{cls+1}}$ by the channel concatenation way. Where $N_{cls+1}$ is equal to the total number of object classes in a given dataset plus 1. The new feature representation $F_s$ is then fed to a patch embedding layer, thus obtaining a sequence embedding $e_1 \in \mathbb {R}^{\frac{W}{4} \times \frac{H}{4} \times 96}$ as the input of the Swin Transformer module. More specifically, the patch embedding layer exploits non-overlapping convolution to partition the feature map $F_s$ into a series of patch tokens with size 4, and then each patch token is flattened into a sequence embedding $e_i$ by linear mapping. Compared to the default $16\times 16$ patch setting, a smaller patch size facilitates to extract the local features containing more detailed information, but also leads to an extended computational workload. The above mapping process from an input semantic layout map to a one-dimensional sequence embedding is summarized as follows:

1 $$\begin{aligned}&F_s = \text {Concat}(\text {Encoding}(M),\text {Candy}(M)), \end{aligned}$$

2 $$\begin{aligned}&e_i = \text {Linear}(\text {Conv}(F_s))), \end{aligned}$$

where Concat, Encoding and Candy denote the concatenation operation, the one-hot encoding operation and the candy edge extraction operation, respectively. And Conv and Linear are utilized to realize patch partition and linear mapping in the patch embedding layer.

The backbone network of the encoder comprises four combinations of the Swin Transformer module and the patch merging layer, which aims to hierarchically extract the fine-grained features at different scales. Specifically, the patch merging layer mainly focuses on downsampling the feature map to influence the dimensions of width, height and number of channels. And the Swin Transformer module mainly focuses on extracting the low-level position features. Each Swin Transformer module $T_i$ is made of two consecutive Transformer blocks based on Layer Normalization (LN)^42^ layer, Multi-head Self-Attention (MSA) layer, Feed-forward Network (FFN) layer, and skip connections, respectively. The two successive Transformer blocks have the same structure, but the MSA layer can be further subdivided into window-based MSA and shift-window-based MSA based on the window division scheme. In the $T_i$ module, the detailed computation of the Transformer block is as follows:

3 $$\begin{aligned}&z_i = \text {MSA}(\text {LN}(e_i)) + e_i, \end{aligned}$$

4 $$\begin{aligned}&e_{i+1} = \text {FFN}(\text {LN}(z_i)) + z_i, \end{aligned}$$

here $e_{i+1}$ represents the output of the Transformer block with a sequence embedding $e_i$ as input. Multi-head Self-Attention (MSA) has three attention heads with parallel and independent computation, which can effectively shorten the computation workload. The specific computing for the MSA is illustrated as follows:

5 $${\text{ESA}}(Q,K,V) = {\text{Softmax}}(\frac{Q\cdot K^{\rm T}}{\sqrt{d_K}}+Bs)V,$$

where $\text {ESA}(Q,K,V)$ Multiple self-attention ESA represents the output from the integration of QKV’s attention. The symbols Q, K and V denote a query vector, a key vector and a value vector respectively. They are obtained by linear mapping of the same input vector $\text {LN}(e_i)$. And, $d_{K}$, *B* and *T* represent the scaled dot-product attention, bias vector and the transpose operation, respectively.

**Figure 3:**
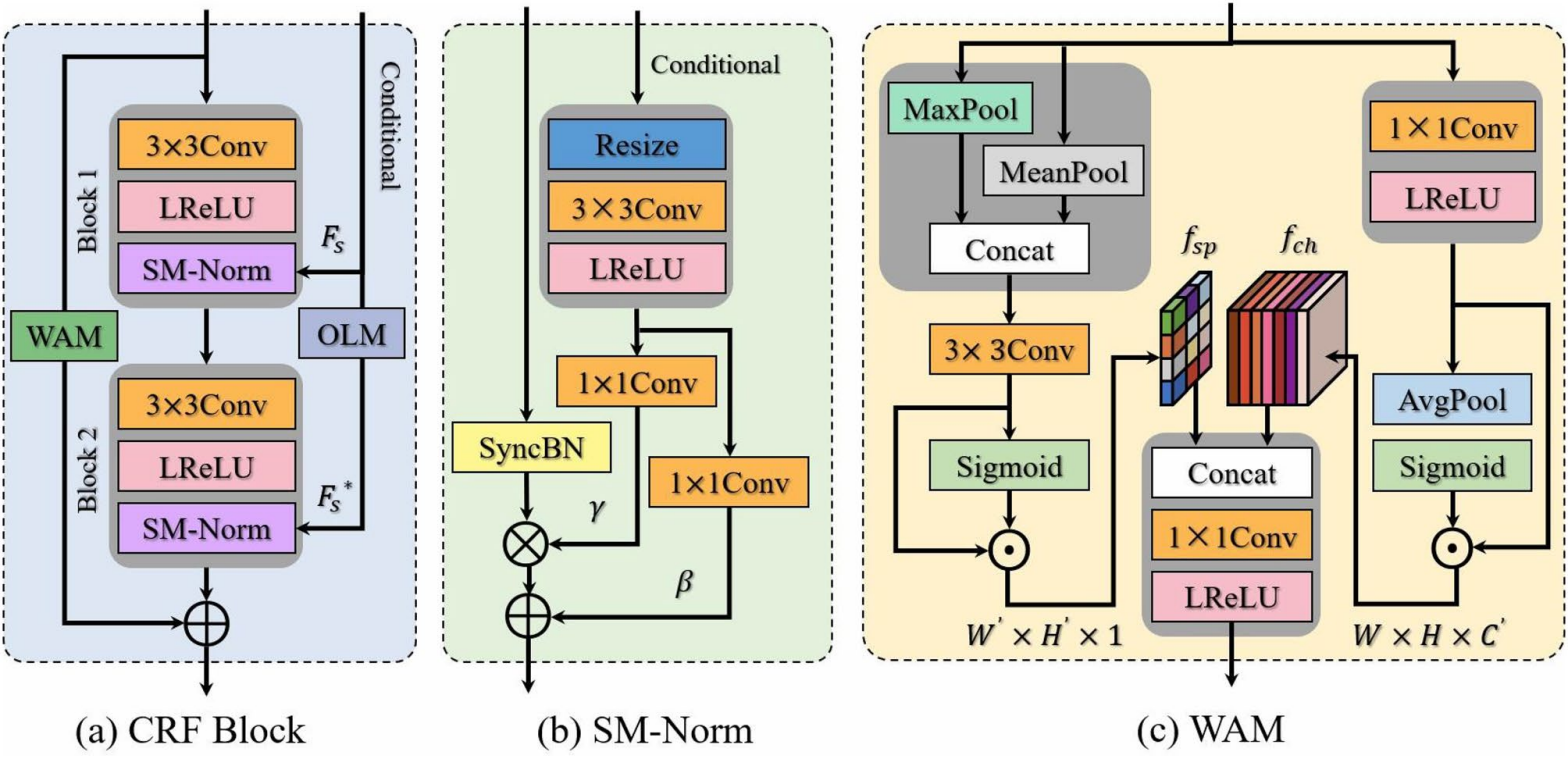
Structure of the CRF block in the SC-UNet method. (**a**) The CRF block represents the conditional residual fusion block. (**b**) SM-Norm denotes the normalization based on semantic modulation. (**c**) WAM stands for the weighting assignment mechanism.

### Model decoder

The decoder will fuse the multi-scale features hierarchically extracted by the encoder from the input semantic layout map to recover a realistic synthesized image at the original resolution size. The hierarchical features at the smallest scale are first passed through a Conditional Residual Fusion (CRF) block based on CNN to obtain high-level features with more semantic information. In order to be concatenated with the low-level features output from the encoder in the channel direction, the obtained high-level features need to be up-sampled to $2 \times$ resolution utilizing a patch expand layer. Subsequently, the fusion results obtained from the high- and low-level features using the concatenation operation performs a CRF block conditional on the feature representation $F_s \in \mathbb {R}^{H \times W \times N_{cls+1}}$ before being fed to the Swin Transform module. Compared with CNN, a series of combining operations constructed with the Swin Transformer module as the backbone can better capture the contextual feature mapping, which incorporates the comprehensive information of the low-level positions and the high-level semantics. Finally, the feature mapping output by Swin Transformer module will pass through an Image Block to recover a naturalistic synthesized image with the $H\times W\times 3$ dimensions. The image block, as the final layer of the decoder, is composed of two CRF blocks, a $3\times 3$ convolution with a padding size of 1, an upsampling function, and a tanh activation function. The following is a detailed description of the CRF block in the decoder network.

### Conditional residual fusion block

Earlier approaches for semantic image synthesis primarily specialized in the extraction of low-level features at multiple scales, while neglecting the information fusion among high- and low-level features. These methods exploit the concatenation in the number of channels to form the thicker features, which are then depended to recover the high quality synthesized image. Motivated by ResNet^36^, we design a Conditional Residual Fusion (CRF) block to achieve more effective information fusion between high- and low-level features at multiple scales. As shown in Fig. 3a, the CRF block is composed of two successive convolutional blocks, a Opposition-based Learning Mechanism (OLM), and a Weight Assignment Mechanism (WAM).

For each convolutional block, our CRF block not only expands the single $3\times 3$ convolution layer by adding a LReLU^22^ activation function and a SM-Norm layer to effectively prevent network overfitting, but it also introduces novel mechanisms to enhance feature extraction. Where SM-Norm stands for the normalization based on semantic modulation, which can effectively improve the convergence speed by shortening the feature differences. Different from the Batch Normalization (BN)^43^, SM-Norm performs the normalization of input activation conditional on $F_s \in \mathbb {R}^{H \times W \times N_{cls+1}}$, and its structure is given in Fig. 3b. The input activation $h \in \mathbb {R}^{H \times W \times C}$ to the SM-Norm layer is first parameter-free normalized along the batch dimension exploiting the Synchronized Batch Normalization (SyncBN). Then, the condition $F_s$ as another input of SM-Norm layer will perform a combined block of Resize-Conv-LReLU to extract the semantic features, and utilizes two $1\times 1$ convolution layers to produce the normalization parameters $\gamma \in \mathbb {R}^{H \times W \times C}$ and $\beta \in \mathbb {R}^{H \times W \times C}$, respectively. Finally, the produced $\gamma$ and $\beta$ are multiplied and added to the normalized activation in the element-wise way. Formally, the SM-Norm layer can be defined as:

6 $$\begin{aligned}&\text {SM-Norm}\left( h, F_s \right) = \frac{h - \mathbb {E}\left[ h \right] }{\sqrt{{\text {Var}}\left( h\right) +\varepsilon }} \cdot \gamma \left( F_s\right) + \beta \left( F_s\right) , \end{aligned}$$

where $\mathbb {E}\left[ h \right]$ and ${\text {Var}}\left( h\right)$ represent the mean and standard deviation of the input activation *h*, respectively. And the symbol $\varepsilon$ usually denotes a very small positive number. The $\gamma$ and $\beta$ learned from condition $F_s$ are used to modulate the normalized activation in scale and bias.

In addition, the CRF block embeds two novel mechanisms, WAM and OLM, to enhance the hierarchical fusion of high- and low-level features. The WAM added on the constant shortcut connection can adaptively assign different attention weights for the input features, thus obtaining effective feature representations enhanced in both channel and spatial dimensions. Due to the sparse semantic information in condition $F_s$, the designed OLM is utilized to actualize the enhancement of semantic feature information.

### Opposition-based learning mechanism

The condition $F_s$ obtained from the input semantic layout map is used to positively influence the normalization layer of CRF block. Accordingly, it is necessary to augment the semantic information of the sparse $F_s$ for the improvement of normalization performance. Computational intelligence employs opposition-based learning^38^, which has been demonstrated to be an efficient way to improve different optimization methods. To augment semantic information in the condition $F_s$, we suggest a fresh Opposition-based Learning Mechanism (OLM) for accomplishing the modulation of the normalization layer.

The condition $F_s$ is gained by the channel concatenation of one-hot label *M* and edge map *E*. Since the semantic information of the condition $F_s$ is mainly derived from the one-hot label *M*, which is the output of performing a one-hot encoding operation on the semantic layout map. Thus, the semantic augmentation result of condition $F_s$ passing through an opposites-based learning mechanism can be expressed as follows:

7 $$\begin{aligned}&F_s^*=\text {Concat}\left( M^*, E\right) ,&\end{aligned}$$

where $M^*$ denotes the opposition-based one-hot label. The central idea underlying opposition-based learning is that the opposing side of a solution is possibly closer to the optimal solution. Let $M = \left\{ m_1 ,m_2 , ... , m_C \right\} ^{H\times W\times C}$ be the one-hot label with multi-channel feature maps. Where the symbols *W*, *H*, and *C* represent the width, height, and number of channels in a semantic condition, respectively. $m_i \in \left\{ a,b\right\} ^{H\times W\times 1}$ denotes a feature map of the *i*th channel. Since each pixel value identifies the object class to which it belongs. In $m_i$, only the pixel value of the *i*th object class is 1, and the other pixels are 0. Referring to the description of the opposite point in opposition-based learning, the opposition-based one-hot label $M^*$ is described as:

8 $$\begin{aligned}&M^* = \left\{ m_1^*,m_2^*,...,m_C^* \right\} ^{H\times W\times C}, \end{aligned}$$

9 $$\begin{aligned}&m_i^*=a+b-m_i, \end{aligned}$$

where, according to the definition of the one-hot label, the thresholds a and b are set to 0 and 1 respectively. ${m_i}^* \in \left\{ 0,1 \right\} ^{H\times W \times 1 }$ represents a feature map of the *i*th channel in the opposition-based one-hot label.

### Weight assignment mechanism

The distribution of redundant information in input features is usually different. Therefore, we design a Weight Assignment Mechanism (WAM) embedded in the shortcut connections, which can adaptively assign different attention weights to the input features. The detailed structure of WAM is presented in Fig. 3c. WAM first extracts important semantic and positional features to filter the redundant information in the input features. And then, the extracted important features are efficaciously fused to output a more powerful feature representation.

The extraction of important semantic features relies on the learning of semantic correlations on the channel dimensions. Since the sub-feature maps on each channel dimension contain different amounts of semantic information, assigning an attention weight to them can extract enhanced semantic features. The input feature $f_{in} \in \mathbb {R}^{H\times W \times C}$ of WAM is first passed through a $1\times 1$ convolution layer and an LReLU activation to produce the intermediate feature $t_{ch} \in \mathbb {R}^{H\times W \times {C}'}$. Then, $t_{ch}$ utilizes an adaptive average pooling layer and a sigmoid activation function to obtain channel attention weights $W_{ch} \in \mathbb {R}^{1\times 1\times {C}'}$, which reflect the importance of the sub-feature maps on each channel dimension. Finally, $t_{ch}$ and $W_{ch}$ are fused using element-wise multiplication to extract the attended semantic features $f_{sc} \in \mathbb {R}^{H\times W \times {C}'}$. This description of the above process can be defined as:

10 $$\begin{aligned}&t_{sc} = \text {LReLU}\left( \text {Conv} \left( f_{in} \right) \right) , \end{aligned}$$

11 $$\begin{aligned}&w_{sc} = \text {Sigmoid} \left( \text {AvgPool} \left( t_{sc}\right) \right) ,\end{aligned}$$

12 $$\begin{aligned}&f_{sc} = t_{sc} \odot w_{sc},&\end{aligned}$$

where $\odot$ denotes element-wise multiplication to fuse the feature information.

Extracting important positional features depends on learning to correlate positions in space. Similarly, each pixel in the spatial location is assigned an attention weight, which helps to extract the enhanced positional features. To learning the spatial relationship, the input feature $f_{in} \in \mathbb {R}^{H\times W \times C}$ first combines the outcomes from the max pooling and average pooling layers in the channel concatenation way to produce a higher dimensional feature $t_{sp} \in \mathbb {R}^{H\times W \times 2}$. To reduce the number of channels, $t_{sp}$ exploits a $3\times 3$ convolution layer with padding size 1, resulting in the intermediate feature ${t^*}_{sp} \in \mathbb {R}^{H\times W \times 1}$. After that, ${t^*}_{sp}$ utilizes a sigmoid activation function to get the spatial attention weights $W_{sp} \in \mathbb {R}^{H\times W\times 1}$, which reflect the importance of the position-wise pixel in the spatial dimension. Finally, we perform a matrix multiplication between ${t^*}_{sp}$ and $W_{sp}$ to extract the attended positional features $f_{sp} \in \mathbb {R}^{H\times W \times 1}$. Mathematically,

13 $$\begin{aligned}&t_{sp} = \text {Concat}\left( \text {MaxPool} \left( f_{in} \right) , \text {MeanPool} \left( f_{in} \right) \right) , \end{aligned}$$

14 $$\begin{aligned}&t^*_{sp} = \text {Conv} \left( t_{sp}\right) , \end{aligned}$$

15 $$\begin{aligned}&w_{sp} = \text {Sigmoid} \left( t^*_{sp} \right) , \end{aligned}$$

16 $$\begin{aligned}&f_{sp} = t^*_{sp} \odot w_{sp} . \end{aligned}$$

The semantic and location features are fused using channel concatenation , and the convolution layer and LReLU activation function will be sequentially performed to generate the final output feature $f_{out} \in \mathbb {R}^{H\times W \times {C}'}$ of the WAM.

**Table 1:** The size change of an original resolution image after being fed into the PatchGAN discriminator.

Operation	Input	Size	Output	Size
ConvLayer$_1$	image	(3,256,256)	$down_1$	(64,128,128)
ConvLayer$_2$	$down_1$	(64,128,128)	$down_2$	(128,64,64)
ConvLayer$_3$	$down_2$	(128,64,64)	$down_3$	(256,32,32)
ConvLayer$_4$	$down_3$	(256,32,32)	$down_4$	(512,16,16)
ConvLayer$_5$	$down_4$	(512,16,16)	$down_5$	(256,16,16)
ConvLayer$_6$	$down_5$	(256,16,16)	out	(1,16,16)

ConvLayer$_i$ stands for the convolution block in the *i*th layer.

**Figure 4:**
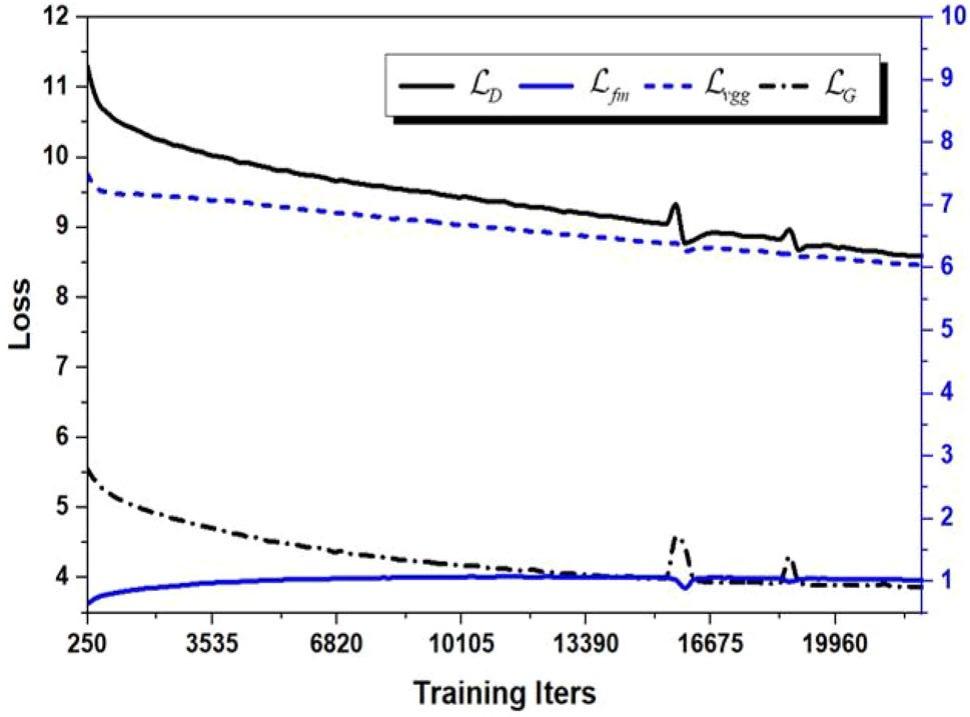
Variation trend of the discriminator and generator loss values with number of iterations during training on the Cityscapes dataset. The black and blue curves indicate the results of total loss and its correlated loss in training, respectively.

### Discriminator and loss function

Similar to GauGAN^22^, we use an efficient multi-scale discriminator, which will perform the adversarial training with our SC-UNet network (also regarded as a generator). The multi-scale discriminator utilizes the integration of multiple PatchGAN discriminators with the same structure, and the input image size is different for each PatchGAN^33^ discriminator. To distinguish between synthesized and real images, the multi-scale discriminator first scales the input image into different sizes and feeds them into the corresponding PatchGAN discriminator. After that, the output matrices of all PatchGAN discriminators are calculated the mean value. Finally, the summation result of the mean value will be applied as the basis for true or false discrimination. In our experiments, the multi-scale discriminator actually uses only two PatchGAN discriminators, and the size of their input images is the original resolution and half of the original resolution, respectively. Table 1 shows the size change of an original resolution image after being fed to the PatchGAN discriminator. Where each PatchGAN discriminator consists of 6 convolution blocks, which is based on the convolution layer, the instance normalization^44^, and the LReLU activation function.

The multi-scale discriminator is optimized using only the hinge-based adversarial loss $\mathscr {L}_{hadv}^{D}$^45^ to distinguish between synthesized and real images. However, the generator is optimized with the weighted sum of the multiple loss functions, which include hinge-based adversarial loss $\mathscr {L}_{hadv}^G$, feature matching loss $\mathscr {L}_{fm}$^30^, and perceptual loss $\mathscr {L}_{vgg}$^30^. Finally, all the above losses are integrated to define the overall optimization goal of the discriminator and generator as,

17 $$\begin{aligned}&\mathscr {L}_D = \mathscr {L}_{hadv}^{D}, \end{aligned}$$

18 $$\begin{aligned}&\mathscr {L}_G = \gamma _{hadv} \mathscr {L}_{hadv}^G + \gamma _{fm} \mathscr {L}_{fm} + \gamma _{vgg} \mathscr {L}_{vgg} , \end{aligned}$$

where $\gamma _{hadv}$, $\gamma _{fm}$, and $\gamma _{vgg}$ denote the weights corresponding to the losses, and set $\mathscr {L}_{hadv}=1$, $\mathscr {L}_{fm}=10$ and $\mathscr {L}_{vgg}=10$ in our experiments.

Figure 4 shows the variation trend of the discriminator and generator loss values with number of iterations during training on the Cityscapes dataset. Where the black and blue curves indicate the results of total loss and its correlated loss in training, respectively. We can observe that the correlated losses from the discriminator and generator are smoothly converging as the number of iterations increases. Moreover, the total losses display a positive correlation with its correlated losses. This indicates that our model mitigates the possibility of over-fitting over the training process owing to the reasonable design of the loss function.

**Figure 5:**
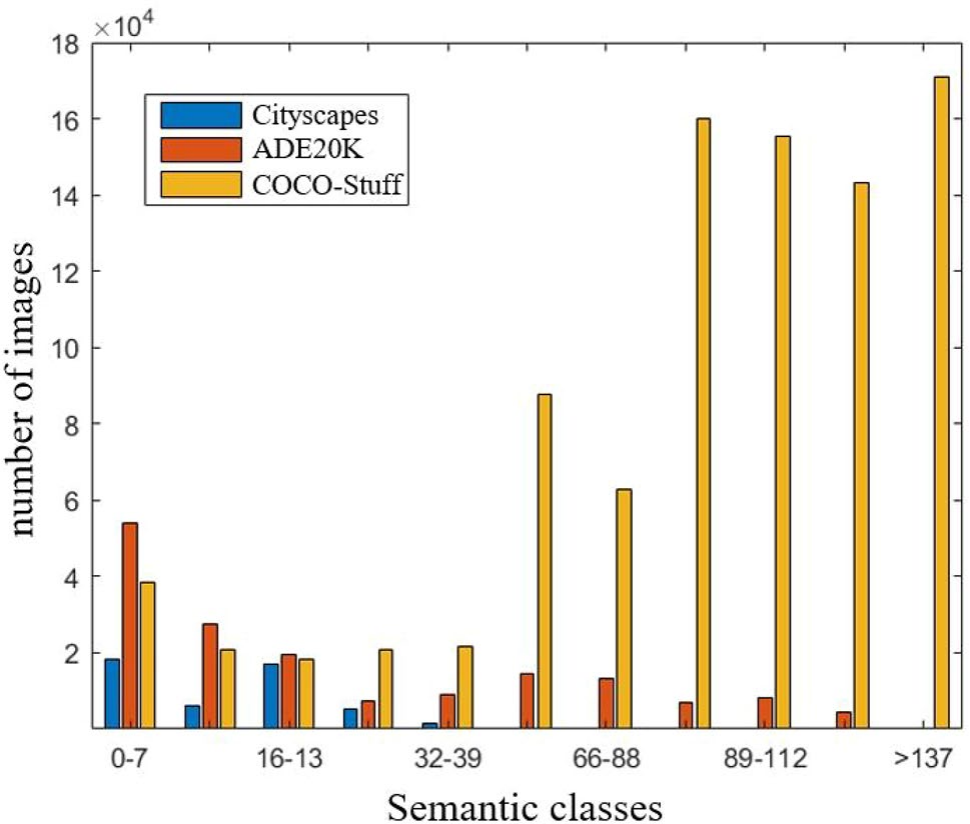
Distribution in the number of images corresponding to each semantic class on the public datasets of Cityscapes, ADE20K and COCO-Stuff.

## Experiments

### Experimental settings

#### Datasets

In order to validate the superiority of the proposed SC-UNet approach, we have carried out extensive experiments on three public datasets: Cityscapes^14^, ADE20K^15^, and COCO-Stuff^16^. The Cityscape dataset includes 35 semantic classes, while training and validation images are 2975 and 500, respectively. The ADE20K dataset has 150 semantic classes, while 20,210 training and 2000 validation images. The COCO-Stuff dataset comprises 182 semantic classes in addition to 118,287 training and 5000 validation images. The distribution in the number of images for each semantic class on the three datasets is displayed in Fig. 5. As can be seen, there is an imbalance in the distribution of semantic categories. In addition, we adjusted the resolutions of the images in the cityscape, ADE20K and COCO-Stuf datasets to $512\times 256$, $256\times 256$ and $256\times 256$, respectively, so as to verify the robustness of the proposed SC-UNet under different image resolutions.

#### Baselines

The baseline models used to implement semantic image synthesis can be broadly classified into unsupervised and supervised. Unsupervised baselines aims to implement a translation of semantic maps to realistic images using unpaired training data. Unsupervised baseline models include CycleGAN^46^, DistanceGAN^47^, MUNIT^48^, DRIT^49^, GCGAN^50^, CUT^51^, USIS^52^ and so on. In contrast, supervised baselines can produce higher quality images by utilising input data with labels. In supervised baseline models, the earlier CRN^29^ and SIMS^35^ are trained without using adversarial training. However, the GAN-based supervised baselines can be further subdivided into other^53–56^, normalization^22,56–61^, attention^7,8,23,31,62,63^, and discriminator^30,32,34,64^ according to the improvement direction.

#### Evaluation metric

Referring to previous work, we adopts both the Fréchet Inception Distance (FID)^65^ as image generation score to assess the perceptual quality and diversity of the synthesized images. Moreover, we also utilize the mean Intersection over Union (mIoU)^29^ and the pixel Accuracy (Acc)^22^ as semantic segmentation scores to measure the segmentation accuracy. We use the state-of-the-art segmentation networks for each dataset: DRN-D-105^66^ for Cityscapes, UperNet101^67^ for ADE20K, and DeepLabV2^68^ for COCO-Stuff.

**Table 2:** Quantitative comparison of our method with the supervised baselines in image generation score (FID) and semantic segmentation scores (mIoU and Acc) on all the datasets.

Method	Cityscapes			ADE20K			COCO-Stuff		
	FID$\downarrow$	mIoU$\uparrow$	Acc$\uparrow$	FID$\downarrow$	mIoU$\uparrow$	Acc$\uparrow$	FID$\downarrow$	mIoU$\uparrow$	Acc$\uparrow$
CRN^29^	104.7	52.4	77.1	73.3	22.4	68.8	70.4	23.7	40.4
SIMS^35^	49.7	47.2	75.5	n/a	n/a	n/a	n/a	n/a	n/a
BicycleGAN^53^	87.7	23.3	75.4	87.8	4.78	29.6	n/a	n/a	n/a
PIS^54^	96.4	64.8	82.4	n/a	n/a	n/a	28.8	38.6	69.0
BatchGAN^55^	73.3	n/a	70.4	49.8	n/a	66.8	n/a	n/a	n/a
SESAME^56^	54.2	66.0	82.5	31.9	49.0	85.5	29.2	n/a	n/a
GauGAN^22^	71.8	62.3	81.9	33.9	38.5	79.9	22.6	37.4	67.9
TSIT^57^	59.2	65.9	82.7	31.6	38.6	80.8	n/a	n/a	n/a
DSCGAN^58^	67.7	37.8	86.7	83.9	10.2	58.8	n/a	n/a	n/a
GroupDNet^59^	49.8	62.3	93.7	42.1	30.4	77.1	n/a	n/a	n/a
CLADE^61^	50.6	60.4	93.4	30.4	35.4	77.3	29.1	36.7	68.0
SelectionGAN^62^	65.2	63.8	82.4	33.1	40.1	81.2	n/a	n/a	n/a
DAGAN^63^	60.3	66.1	82.6	31.9	40.5	81.6	n/a	n/a	n/a
LGGAN^31^	57.7	68.4	83.0	31.6	41.6	81.8	n/a	n/a	n/a
SC-GAN^23^	49.5	66.9	82.5	29.3	45.2	83.8	18.1	42.0	72.0
SelectionGAN$\dagger$^7^	63.4	64.5	82.7	32.2	41.7	81.5	n/a	n/a	n/a
LGGAN$\dagger$^8^	48.1	67.7	82.9	30.5	41.4	81.5	n/a	n/a	n/a
Pix2PixHD^30^	95.0	58.3	81.4	81.8	20.3	69.2	111.5	14.6	45.7
CC-FPSE^34^	54.3	65.6	82.3	31.7	43.7	82.9	19.2	41.6	70.7
OASIS^32^	47.7	69.3	n/a	28.3	48.8	n/a	17.0	**44.1**	n/a
SAFM^64^	49.5	70.4	83.1	32.8	50.1	**86.6**	24.6	43.3	73.4
SC-UNet(Ours)	**44.9**	**70.6**	**94.4**	**27.8**	**51.4**	85.7	**16.5**	44.0	**73.6**
	+2.8	+0.2	+0.7	+0.5	+1.3	-0.9	+0.5	-0.1	+0.2

“n/a” indicates that the visual result is not provided on the official website of the model. The boldface denotes the best performance.

#### Implementation details

We utilize the ADAM optimizer^69^ with $\beta _1=0$ and $\beta _2=0.999$ to train our models on a single RTX 3090Ti GPU. The learning rates of the generator and the discriminator are defined as *lr*/2 and $lr * 2$, where the initial value of the learning rate *lr* is set to 0.0002. To more accurately find the global optimal solution, the learning rate is dynamically changed during the training process. Formally, the dynamic learning rate is represented as follows:

19 $$\begin{aligned} lr_t={\left\{ \begin{array}{ll} lr, &{} {0 \le t < m} \\ lr - \frac{lr}{n-m} * (t-m) ,&{} { m \le t \le n} \end{array}\right. }, \end{aligned}$$

where *n* is the total number of training epochs and $m=n /2$. According to the above formula, the learning rate will linearly decay to zero after *m* epochs. Furthermore,we train 200 epochs on the cityscape and ADE20K datasets to find the optimal solution, and 100 epochs on the COCO-Stuff dataset due to the large number of training images.

**Table 3:** Quantitative comparison of our method with the unsupervised baselines in image generation score (FID) and semantic segmentation score (mIoU) on three public datasets.

Method	Cityscapes		ADE20K		COCO-Stuff	
	FID$\downarrow$	mIoU$\uparrow$	FID$\downarrow$	mIoU$\uparrow$	FID$\downarrow$	mIoU$\uparrow$
CycleGAN^46^	87.2	24.5	96.3	5.40	104.7	2.08
DistanceGAN^47^	78	17.6	80	0.035	92.4	0.014
MUNIT^48^	84	8.2	n/a	n/a	n/a	n/a
DRIT^49^	164	9.5	132.2	0.016	135.5	0.008
GCGAN^50^	80	8.4	92	0.07	99.8	0.019
CUT^51^	57.3	29.8	79.1	6.9	85.6	2.21
USIS^52^	53.7	44.8	33.2	17.38	27.8	14.06
SC-UNet(ours)	**44.9**	**70.6**	**27.8**	**51.4**	**16.5**	**44.0**
	+8.8	+25.8	+5.4	+34.02	+11.3	+29.94

“$\downarrow$” means lower performance is better. “$\uparrow$” means higher performance is better. “+” represents the amount of improvement.

Significant values are in bold.

**Figure 6:**
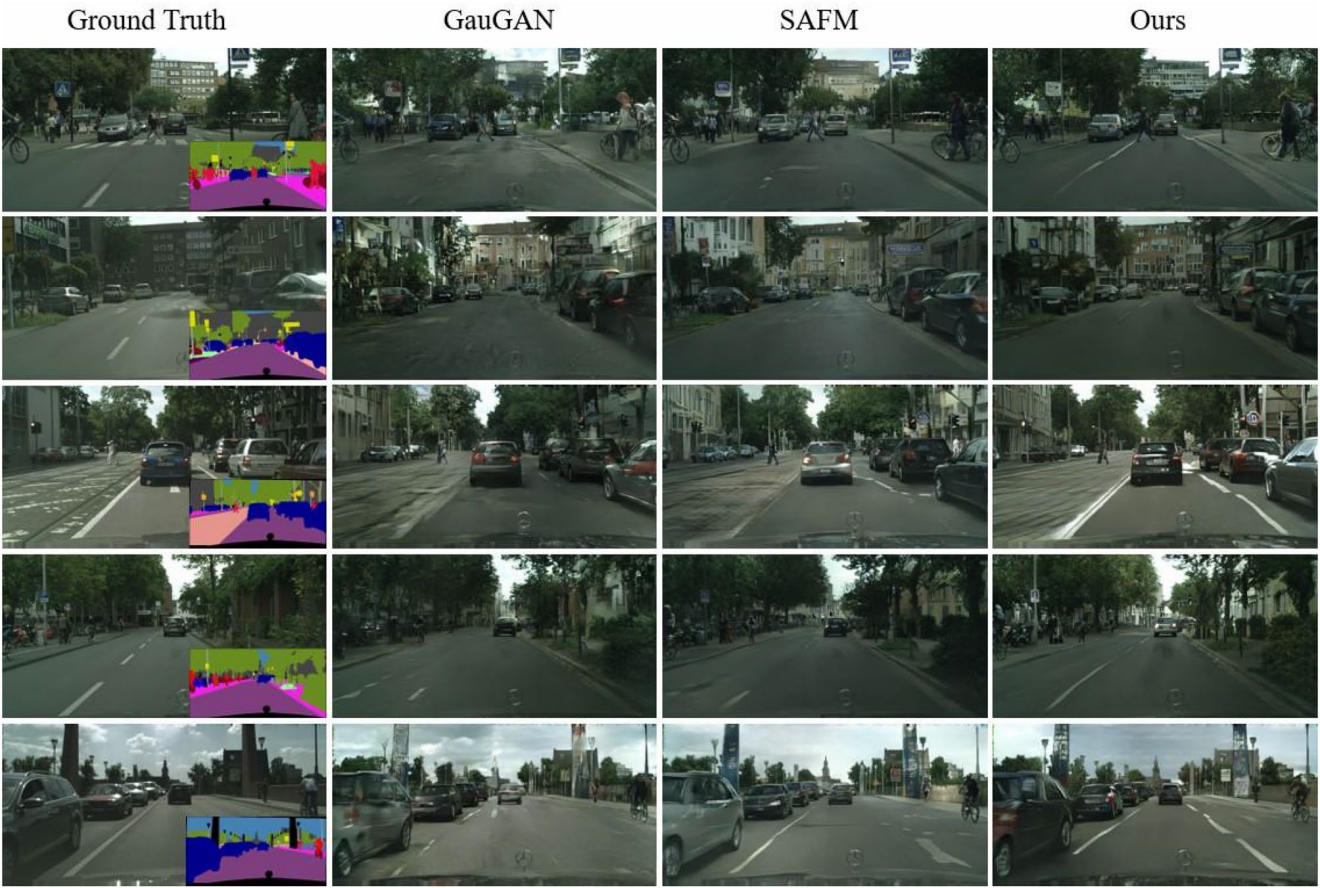
Qualitative comparison of our SC-UNet mothod with the competing methods on Cityscapes dataset. Our method generates images with better visual quality and higher-fidelity details.

**Table 4:** Similarity matching result of the average power spectrogram.

Power spectrograms	Similarity	
	ORB^70^	Histogram^71^
GT vs GauGAN^22^	0.62675	0.70314
GT vs SAFM^64^	0.80372	0.81605
GT vs ours	**0.89701**	**0.88362**

Significant values are in bold.

### Quantitative results

Table 2 gives the quantitative comparison results of our method with the supervised baselines in image generation score (FID) and semantic segmentation scores (mIoU and Acc) on the Cityscapes, ADE20K and COCO-Stuff datasets. The results in the table show that our method obtains a lower generation score (FID) than the previous supervised baselines on the validation set for each dataset. The lower the generation score, the higher the fidelity and diversity of the synthesized images produced by the deep learning network. In addition, our proposed method acquires a higher semantic segmentation scores (mIoU and Acc) than previous state-of-the-art models on the Cityscapes dataset, which has a small data amount and a relatively homogeneous distribution of semantic classes. In order to improve the semantic alignment with the input layout map, the latest OASIS^32^ and SAFM^64^ utilize the idea of semantic segmentation to improve the discriminator network. Although OASIS and SAFM obtain higher Acc and mIoU scores than our approach, this slight improvement only appears in the ADE20K and COCO-Stuff datasets with large data amounts and unbalanced semantic class distributions. Therefore, the quantitative comparison with the baselines confirms the superiority of our proposed network model in semantic image synthesis.

Furthermore, the quantitative comparison of our method with the unsupervised baselines is reported in Table 3. Compared to the unsupervised baselines, we achieve better image generation score (FID) and semantic segmentation score (mIoU) on three public datasets by constructing a supervised model. Our improvement in the semantic segmentation score is particularly significant, mainly due to the supervised learning under the input semantic layouts. Moreover, the large amount of improvement indicates that the supervised strategy is more beneficial for the semantic image synthesis task.

**Table 5:** Human perceptual evaluation.

Method	Dataset		
	Cityscapes (%)	ADE20K (%)	COCO-Stuff (%)
Ours > GauGAN^22^	73.02	64.58	80.01
Ours > OASIS^32^	54.10	61.34	50.09
Ours > SAFM^64^	62.48	58.92	53.42

These values reflect the average probability of our method being approved by the workers comparing to the baseline method in image synthesis.

#### Human perceptual evaluation

To further validate that our method performs better in the semantic image synthesis, we perform a human perception evaluation^22,23,56^ to compare our approach with the several baseline methods of GauGAN^22^, DAGAN^63^, OASIS^32^, and SAFM^64^ on the Cityscapes, ADE20K and COCO-Stuff datasets. Specifically, we first randomly select 200 semantic layout mappings from the validation set of each dataset to synthesis images for our method and the competing method. Then, we also randomly select 100 AMT workers to conduct the evaluation. Where AMT (known as Amazon Mechanical Turk^72^) is a crowdsourcing marketplace that allows researchers to outsource their tasks to a distributed worker who can volunteer to perform the task for pay. Therefore, this experiment was carried out in accordance with relevant guidelines and regulations, and was obtained the approval of the AMT institutions, and the informed consent from all AMT workers. In each experiment, workers are required to select the perceptually more photo-realistic image from the shown two groups of synthesized images. The two groups of images are synthesized by our method and a competing method, respectively. Finally, we utilize the conventional statistical operations to obtain the average probability that the images synthesized by our method are selected by the workers on each dataset, and the results are shown in Table 5. The comparison results of the human perception evaluation reaffirm our method, and the images synthesized by it are more acceptable in terms of quality.

**Figure 7:**
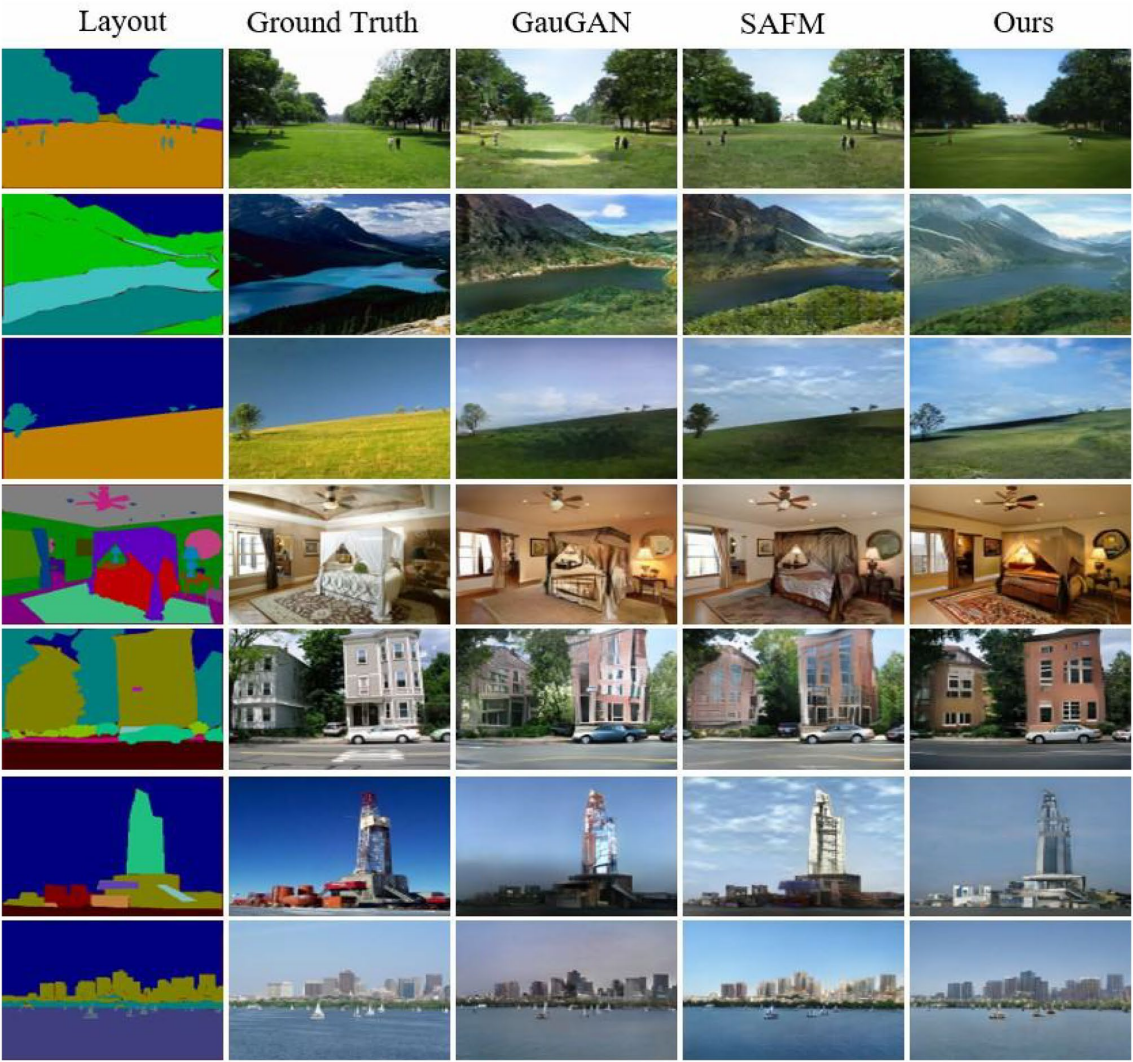
Qualitative comparison results on the ADE20K dataset. Despite diverse semantic classes and small textures, our approach still ensures high fidelity.

**Table 6:** Traditional statistical evaluation.

Method	Statistical evaluation		
	F-statistic^73^	p-value^74^	ANOVA^75^
GauGAN^22^	161.92	1.7492	$\times$
OASIS^32^	173.70	1.0959	$\times$
SAFM^64^	188.39	2.1163	$\times$
Ours	**82.629**	**5.2108**	$\checkmark$

These values reflect the difference between the synthesised image and the real image.

Significant values are in bold.

#### Traditional statistical evaluation

To further emphasize the efficacy of our method in semantic image synthesis tasks, we employed conventional statistical assessment techniques, including F-statistic^73^, p-value^74^, and Analysis of Variance (ANOVA)^75^. As depicted in Table 6, our approach yielded a lower F-statistic of 82.629 and a higher p-value of 5.2108. This observation suggests that, compared to existing unsupervised methods such as GauGAN^22^, OASIS^32^, and SAFM^64^, our method ensures minimal disparities among synthesized image samples. Additionally, ANOVA results indicate no discernible differentiation between the synthesized image dataset and the authentic image dataset, further substantiating the robustness of our approach.

### Qualitative results

**Figure 8:**
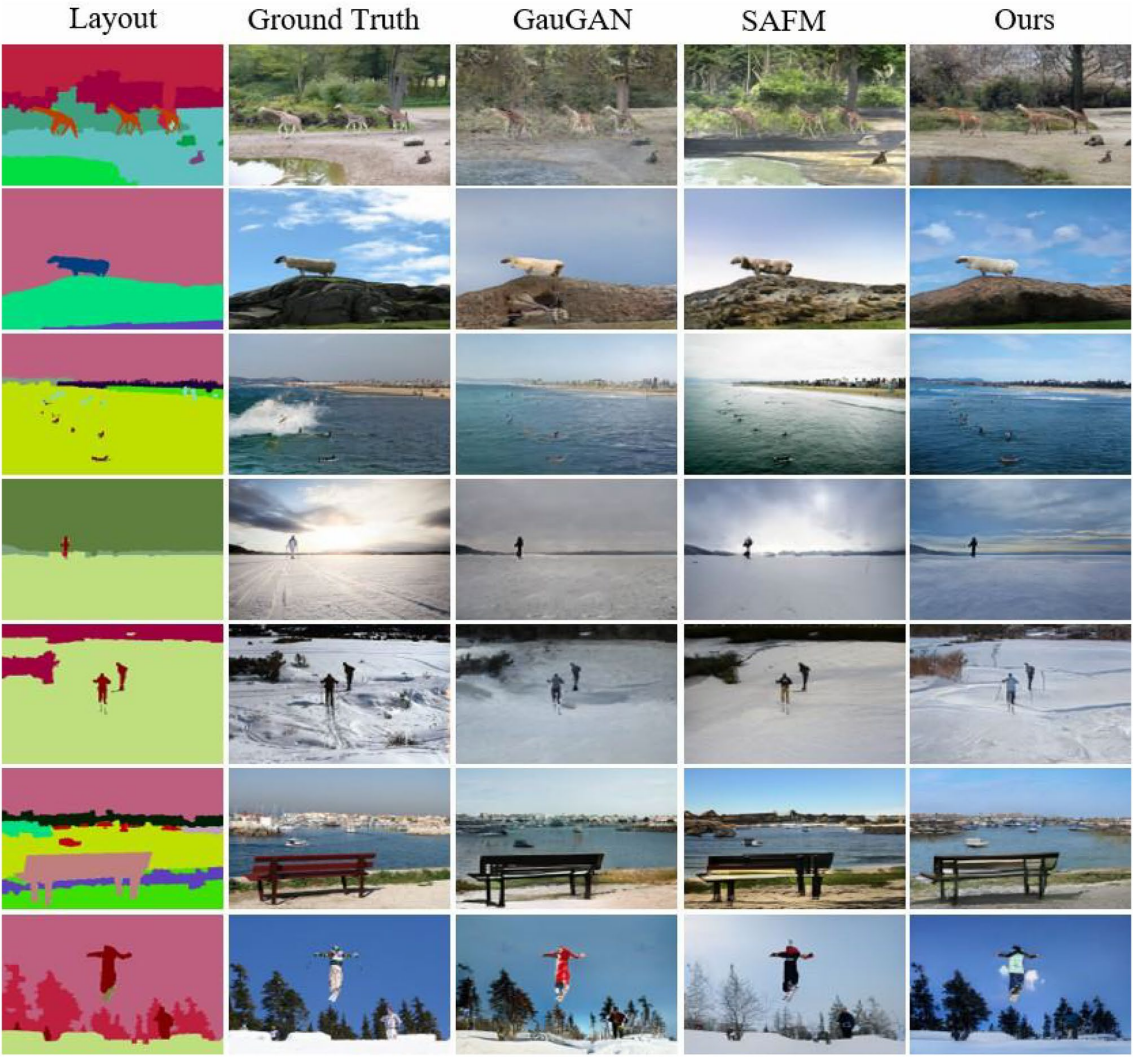
Qualitative comparison results on the COCO-Stuff dataset. The comparison results show that the images synthesized by our model have a higher quality than GauGAN and SAFM.

In Figs. 6, 7 and 8 give the qualitative comparison of our model with the competing methods^22,64^ on Cityscapes, ADE20K and COCO-Stuff datasets. We found that the images synthesized by our model not only have better perceptual quality, but also are closer to the ground truth images in the overall color and texture distribution. Note that the complex real-world scenes synthesized by our method show significant improvement on Cityscapes datasets. However, SAFM^64^ is the current state-of-the-art method, but the images synthesized by it are too bright and even show color distortion. Compared with them, our proposed approach produces photo-realistic images while respecting the input semantic layout map, and can generate challenging scenes with high image fidelity.

#### Mean power spectrogram

**Figure 9:**
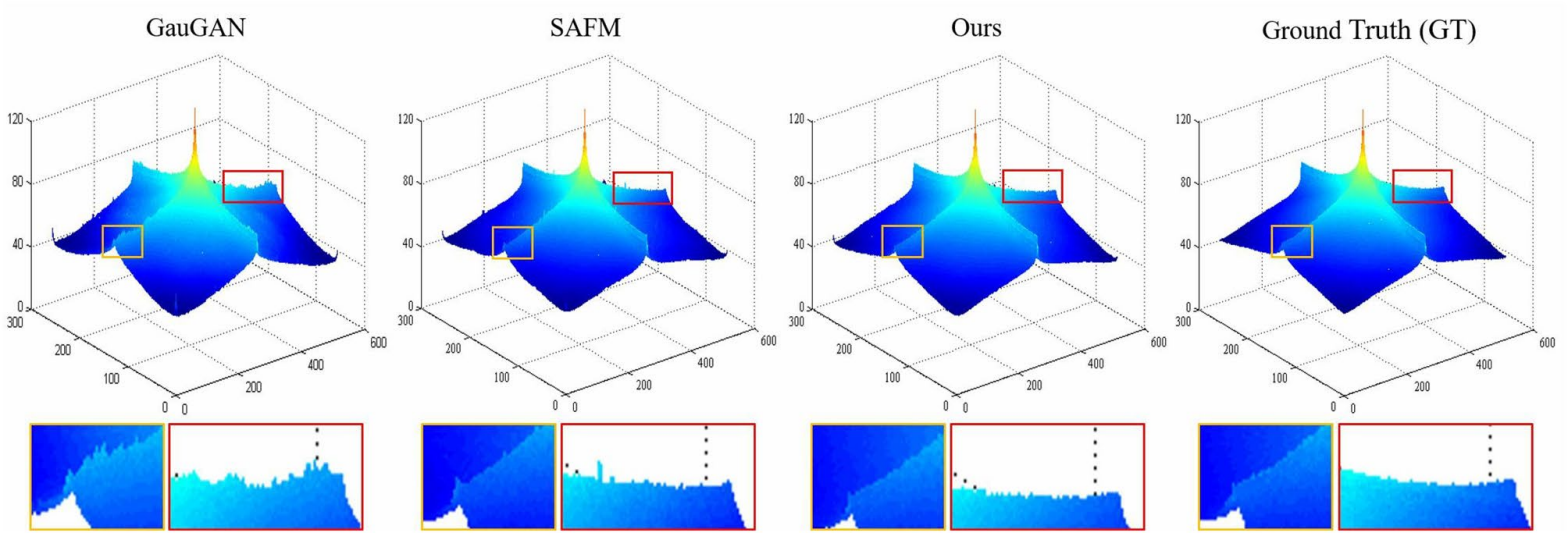
Mean power spectra over the Cityscapes dataset. Key differences are positioned with boxes on the mean power spectra, and shown magnified below image. Magnitude is on a linear scale.

We also calculated the mean power spectrograms of images synthesized by our method with competing methods^22,64^ on the Cityscapes dataset to compare the qualitative from a signal perspective. The similarity matching result of the average power spectrum is shown in Fig. 9. It is intuitively obvious that the two power spectrograms drawn separately from the ground-truth images and the synthesized images produced by our method are the most similar from the perspective of color, texture, and shape. Comparatively, the mean power spectrogram drawn from synthesized images produced by the competing methods showed distinct spikes. Some even present pseudo-local maxima, which are not observed in the average power spectrogram of the ground-truth images. Regarding the differences mentioned above, they can be clearly observed in the comparison of the zoomed-in areas. This enhancement allows for a more detailed examination of the discrepancies. Moreover, we utilize the ORB^70^ and Histogram^71^ algorithm to calculate the similarity between the ground-truth images and images synthesized by our method, and the results are shown in Table 4. Where the higher the value, the more similarity. The similarity matching results calculated by the mean power spectrograms also can validate that the images synthesized by our method are more photo-realistic in details.

### Ablation studies

#### Ablation on important components in SC-UNet

To verify the effectiveness of of each component in our SC-UNet method, we compare our SC-UNet method with three variants on the Citysacpes dataset. These three variants are obtained by gradually replacing or eliminating each component in the framework with our method as a benchmark. Specifically include: (i) “Ours” denotes our proposed SC-UNet model , which is used as a benchmark for the ablation experiments. (ii) “w/o SwinT” denotes that the Swin Transformer (SwinT) module is replaced by the traditional convolutional block to construct a pure CNN-based UNet-like network. (iii) “w/o CRF” represents that the designed Conditional Residual Fusion (CRF) block is replaced by the conventional residual block to fuse the high- and low-level feature information.(iv) “w/o OLM” does not use the designed Opposition-based Learning Mechanism (OLM) to enhance the semantic feature information. (v) “w/o WAM” does not use the Weight Assignment Mechanism (WAM) to allocate attention weights in channel and spatial dimensions. The results of the ablation study are shown in Table 7. By the pair-wise comparison between our SC-UNet method and other variants, we can observe that the SwinT is used as the backbone network to achieve better synthetic performance than pure CNN. Furthermore, it also validates the effectiveness of the CRF block, OLM and WAM components in SC-UNet for high-squality image synthesis based on semantic layout maps. Although the “w/o OLM” method is slightly lower than ours in terms of image synthesis score (FID) , the synthesized images from our SC-UNet approach have better performance in terms of two semantic segmentation scores, mIoU and Acc.

**Table 7:** Ablation studies on important components in SC-UNet.

Method	Metric		
	FID$\downarrow$	mIoU$\uparrow$	Acc$\uparrow$
SC-UNet (Ours)	44.9	**70.6**	**94.4**
w/o SwinT	54.8	67.4	93.7
w/o CRF	48.4	69.1	94.2
w/o OLM	**43.3**	68.9	94.4
w/o WAM	47.8	70.2	94.3

Bold denotes the best performance.

**Table 8:** Ablation studies on discriminator and loss function.

Method	Metric		
	FID$\downarrow$	mIoU$\uparrow$	Acc$\uparrow$
SC-UNet (ours)	**44.9**	**70.6**	**94.4**
PatchGAN	49.1	70.1	94.3
FPSE-D	46.3	68.2	95.2
w/o $\mathscr {L}_{hadv}$	45.0	69.1	94.4
w/o $\mathscr {L}_{fm}$	51.2	69.4	93.8
w/o $\mathscr {L}_{vgg}$	45.8	65.3	94.0

Significant values are in bold.

#### Ablation on discriminator and loss function

Our SC-UNet approach employs adversarial training based on the multi-scale discriminator, which improves the synthesis performance. To highlight the superiority of multi-scale discriminator, we utilize two discriminators available for replacement: a single-scale Markov discriminator^23^ (denoted as “PatchGAN”) and a feature pyramid semantic embedding discriminator^16^ (denoted as “FPSE-D”). As shown in Table 8, our SC-UNet method with aid of multi-scale discriminator not only performs well in terms of semantic segmentation scores (mIoU and Acc), but also excels in terms of image generation scores (FID).

To explore the effect of each loss function on semantic image synthesis, we use the combination of three loss functions as a baseline, and randomly replace or eliminate one of them for each comparison. Specifically, “w/o $\mathscr {L}_{hadv}$” denotes that the hinge-based adversarial loss is replaced by the conditional adversarial loss. “w/o $\mathscr {L}_{fm}$” and “w/o $\mathscr {L}_{vgg}$” represent constraints without the feature matching loss and the perceptual loss, respectively. As shown in Table 8, hinge-based adversarial loss have more obvious advantages than conditional adversarial loss in semantic image synthesis. However, the fundamental difference between feature matching loss and perceptual loss is the image feature extraction network, and the two belong to a dynamic and a static relationship. Compared to the constraints of one loss, the combined effect of two losses can improve the quantitative quality of image synthesis.

**Table 9:** Ablation studies on various image sizes.

$\text {Height}(H)\times \text {Width}(W)$	Metric		
	FID$\downarrow$	mIoU$\uparrow$	Acc$\uparrow$
$256\times 512$	**44.9**	**70.6**	**94.4**
$512\times 1024$	45.2	68.4	91.5
$1024\times 2048$ (original)	50.8	64.2	87.6

Significant values are in bold.

#### Ablation on various image sizes

To explore the impact of image size on synthesis performance, we conducted an ablation study on different image sizes in Table 9. First, images from the Cityscapes dataset were resized to $1024\times 2048$, $512\times 1024$, and $256\times 512$, respectively. Subsequently, we conducted model training by solely controlling the image size as the variable. The results in the table demonstrate that lower resolutions correspond to better synthesis performance. As the image resolution increases, so does the amount of detailed information contained within, necessitating the model to possess a stronger learning capacity for effective processing.

**Figure 10:**
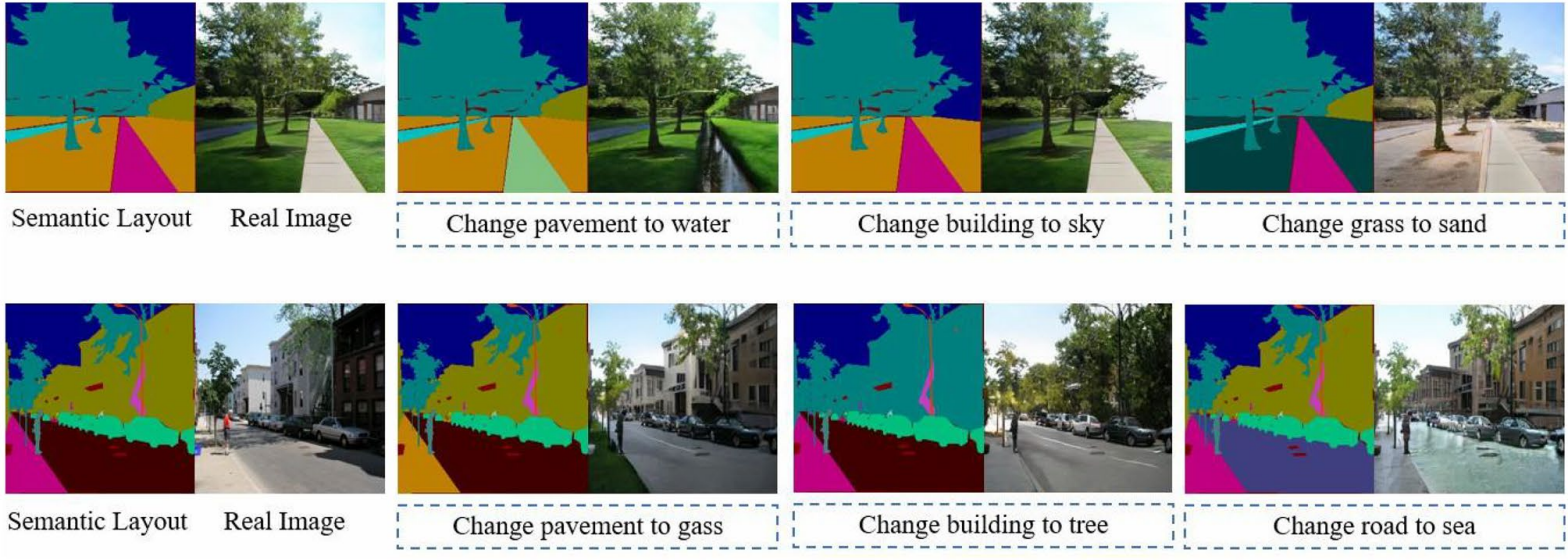
An example application of semantic control synthesis based on our SC-UNet method.

## Conclusion

In this paper, we propose a new semantic image synthesis method (SC-UNet) , which can transform a given semantic layout map into the synthesized images with visual fidelity and semantic alignment. Our SC-UNet model is able to decode more photo-realistic images from the hierarchical feature representations encoded from the input semantic layout maps, by building a U-shaped network using the Swin Transformer module as the basic unit. Furthermore, the skip connection is added to a U-shaped network to combine the high- and low-level features of both sides. To compensate for the loss of semantic information resulted from down-sampling, the low-level features are copied to the high-level features by skip connections. An effective Conditional Residual Fusion (CRF) block is designed to obtain the important semantic and location information from the concatenation of high- and low-level features for higher-quality image synthesis and lower memory usage. The performance improvement of CRF blocks is mainly attributed to the embedding of a opposition-based learning mechanism and a weight assignment mechanism. The opposition-based learning mechanism can effectively enhance the semantic feature information, while the weight assignment mechanism can dynamically assign attentional weights in channel and spatial dimensions. Experimental results show that our proposed method outperforms state-of-the-art methods on three baseline datasets, both qualitatively and quantitatively. Moreover, our SC-UNet method can offer widespread applications, such as content generation and image editing, by adding, deleting, or editing objects. Two examples of applications based on the SC-UNet method are as follows.

### Semantic control synthesis

Figure 10 displays an example application of semantic control synthesis based on our method. In figure, two semantic layout maps for model testing are selected from “ADE_val_00000677.png” and “ADE_val_00000851.png” in ADE20K dataset, respectively. Considering the complexity of manipulating the real scene image, we can change the input semantic layout map from its segmentation to remove or add the objects. Subsequently, the semantic class of a target object is changed, while our model manipulates the real image with the changed semantic layout map. Thus, an ordinary user is also able to interactively manipulate the real image. As can be seen from the results of the semantic control synthesis, our approach can generate realistic and semantically aligned images.

**Figure 11:**
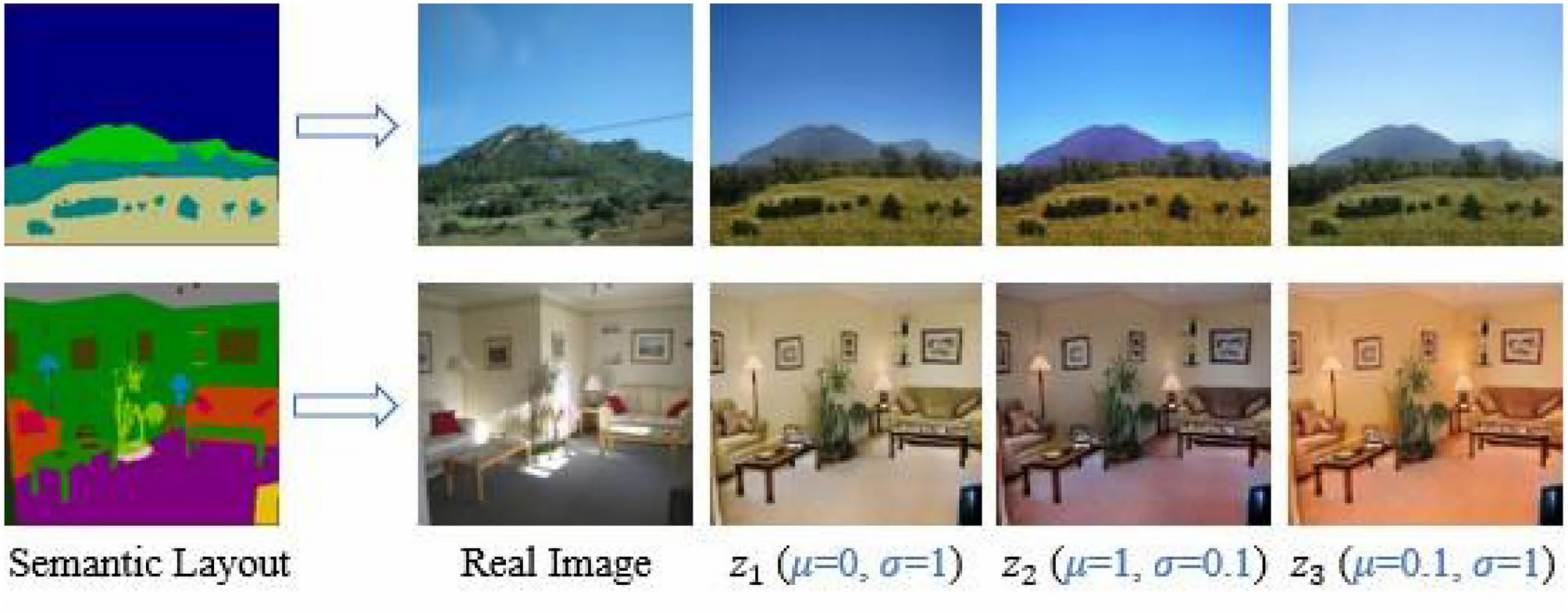
An example application of multi-style image synthesis based on our proposed SC-UNet method. $z_1$, $z_2$ and $z_3$ denote three different random noise tensors, respectively. The symbols $\mu$ and $\delta$ represent the mean and variance of the noise sampling, respectively.

### Multi-style image synthesis

Figure 11 displays an example application of multi-style image synthesis based on our method. In figure, two semantic layout maps for model testing are selected from “ADE_val_00000574.png” and “ADE_val_ 00001512.png” in ADE20K dataset, respectively. We achieve three different styles of image synthesis from the same semantic layout map by randomly sampling of different 3D noise tensor *z*. Our method enables to synthesize different styles of high-fidelity images in indoor and outdoor scenes by noise sampling. The colour, luminance and illumination of the synthesised images can be adjusted, but the semantic structure is basically unchanged.

## Data Availability

Correspondence and requests for data and materials should be addressed to B.C.
